# Exploration of the relationship between gut microbiota and fecal microRNAs in patients with major depressive disorder

**DOI:** 10.1038/s41598-022-24773-7

**Published:** 2022-12-05

**Authors:** Hui-Mei Chen, Yu-Chu Ella Chung, Hsi-Chung Chen, Yen-Wenn Liu, I-Ming Chen, Mong-Liang Lu, Felix Shih-Hsiang Hsiao, Chun-Hsin Chen, Ming-Chyi Huang, Wei-Liang Shih, Po-Hsiu Kuo

**Affiliations:** 1 Institute of Epidemiology and Preventive Medicine, College of Public Health, National Taiwan University, Taipei, 100 Taiwan; 2 Center for Neuropsychiatric Research, National Health Research Institutes, Miaoli, 350 Taiwan; 3 Department of Psychiatry, National Taiwan University Hospital, Taipei, 100 Taiwan; 4 Center of Sleep Disorders, National Taiwan University Hospital, Taipei, 100 Taiwan; 5 Institute of Biochemistry and Molecular Biology, National Yang Ming Chiao Tung University, Taipei, 112 Taiwan; 6 Institute of Health Policy and Management, College of Public Health, National Taiwan University, Taipei, 100 Taiwan; 7 Department of Psychiatry, Wan Fang Hospital, Taipei Medical University, Taipei, 116 Taiwan; 8 Department of Psychiatry, School of Medicine, College of Medicine, Taipei Medical University, Taipei, 110 Taiwan; 9 Department of Biotechnology and Animal Science, National Ilan University, No. 1, Sec. 1, Shennong Rd., Yilan City, Yilan County, 260007 Taiwan; 10 Department of Psychiatry, Taipei City Psychiatric Center, Taipei City Hospital, Taipei, 110 Taiwan; 11 Infectious Diseases Research and Education Center, Ministry of Health and Welfare and National Taiwan University, Taipei, 100 Taiwan; 12 Psychiatric Research Center, Wan Fang Hospital, Taipei Medical University, Taipei, Taiwan

**Keywords:** Genetics research, Depression, Bacterial genetics

## Abstract

Microbiota-gut-brain axis signaling plays a pivotal role in mood disorders. The communication between the host and the gut microbiota may involve complex regulatory networks. Previous evidence showed that host-fecal microRNAs (miRNAs) interactions partly shaped gut microbiota composition. We hypothesized that some miRNAs are correlated with specific bacteria in the fecal samples in patients with major depressive disorder (MDD), and these miRNAs would show enrichment in pathways associated with MDD. MDD patients and healthy controls were recruited to collect fecal samples. We performed 16S ribosome RNA sequence using the Illumina MiSeq sequencers and analysis of 798 fecal miRNAs using the nCounter Human-v2 miRNA Panel in 20 subjects. We calculated the Spearman correlation coefficient for bacteria abundance and miRNA expressions, and analyzed the predicted miRNA pathways by enrichment analysis with false-discovery correction (FDR). A total of 270 genera and 798 miRNAs were detected in the fecal samples. Seven genera (*Anaerostipes*, *Bacteroides*, *Bifidobacterium*, *Clostridium*, *Collinsella*, *Dialister*, and *Roseburia*) had fold changes greater than one and were present in over 90% of all fecal samples. In particular, *Bacteroides* and *Dialister* significantly differed between the MDD and control groups (p-value < 0.05). The correlation coefficients between the seven genera and miRNAs in patients with MDD showed 48 pairs of positive correlations and 36 negative correlations (p-value < 0.01). For miRNA predicted functions, there were 57 predicted pathways with a p-value < 0.001, including MDD-associated pathways, axon guidance, circadian rhythm, dopaminergic synapse, focal adhesion, long-term potentiation, and neurotrophin signaling pathway. In the current pilot study, our findings suggest specific genera highly correlated with the predicted miRNA functions, which might provide clues for the interaction between host factors and gut microbiota via the microbiota-gut-brain axis. Follow-up studies with larger sample sizes and refined experimental design are essential to dissect the roles between gut microbiota and miRNAs for depression.

## Introduction

Major depressive disorder (MDD) presents a significant public health issue, with more than 163 million people affected^1^. As MDD is debilitating itself, it also increases the risk of developing other medical conditions, such as diabetes mellitus, heart diseases, and severe cognitive impairment^2^. In particular, depressed individuals commonly reported gastrointestinal dysfunction and disordered bowel habit^3^, indicating the link between the gut and brain health. In this regard, recent work exploring the causal effects of gut microbiota on human health has suggested the interactions between gut microbiota and host microRNAs (miRNAs)^4–6^.

Over the past decade, the knowledge of the roles of gut microbiome in brain development and disease outcomes has significantly been improved^7–10^. The concept of microbiota-gut-brain axis has been established based on preclinical studies, mainly from mice studies for depression^11–13^. However, results yielded by the stress-like and depressive animal models may not apply directly to human studies. It was not until the year 2014 that the first human study used high-throughput sequencing technology to investigate the associations between fecal microbiota and clinical depression^14^. Subsequently, a few studies reported different taxa to be associated with MDD^15,16^. These studies usually had a limited sample size, and their findings are relatively inconsistent for MDD^15,17^. On the other hand, the Flemish Gut Flora Project in a western population cohort, was the first to explore the link between gut microbiota and mental health in the population-level^18^. The authors reported that two genera *Coprococcus* and *Dialiste*r were depleted in depressed individuals. They further found that 56 biochemical metabolites produced by gut microbiota might affect nervous system functions, which have been reported to be closely related to mood dysregulation and suicidal behaviors^18^.

MicroRNAs (miRNAs), small single-stranded RNAs of approximately 22 nucleotides in length, play vital roles in regulating gene expression at the post-transcriptional level, and are highly involved in the development of certain human diseases^19–23^. Using fluid samples such as blood and cerebrospinal fluid or brain-associated tissues, such as the cortex and basolateral amygdala, the associations between miRNAs and depression in humans have been reported in a review article^24^, despite lacking robust findings to serve as potential biomarkers for depression. Recently, an autism-associated study using fecal samples found that autistic patients with gut dysbiosis were also associated with transcriptional modulators, which were involved in intestinal permeability and inflammation^6^. However, as far as we know, there are no studies exploring miRNAs' roles in fecal samples for depression. The human gut is an ecosystem consisting of eukaryotic and prokaryotic cells. An early study has shown that some specific miRNAs had significant differential expressions within the ileum and the colon between germ-free mice and colonized mice^25^. Later, it was further found that fecal miRNAs are secreted mainly by intestinal epithelial cells and the homeodomain-only protein-positive cells^26^. Additionally, the presence and abundance of specific colitogenic microbiota, such as Enterobacteriaceae and Helicopter, in mice studies have been associated with fecal miRNAs^27^. Therefore, host-fecal miRNAs interactions might shape the composition of the gut microbiota.

In our previous study, we found specific gut microbiota and biological pathways to differ in patients with MDD compared with healthy controls significantly^15^. Inspired by recent intriguing findings of the crosstalk between gut microbiota and miRNAs, we hypothesized that some miRNAs might be correlated with specific gut bacteria for patients with MDD, and these miRNAs would show enrichment in pathways that are reported to be associated with MDD. To the best of our knowledge, this is the first pilot study to explore the relationship between fecal miRNAs and gut microbiota in patients with MDD.

## Results

### Characteristics of the study samples

The clinical characteristics of our samples are displayed in Table 1. There were no significant differences in age, body-mass-index, or gender between the MDD and control groups. No controls were taken an antidepressant, while 77% of patients with MDD were treated with antidepressants. In addition, higher BAI, BDI, and PSS scores were reported in MDD patients than in controls, though the differences did not reach statistical significance.

**Table 1:** Clinical characteristics of the subjects.

Variables	HC (n = 10)	MDD (n = 10)	p-value
**Demographic characteristics**			
Age (Y), means (SD)	38.20 (15.24)	40.90 (14.86)	0.693
body-mass-index, means (SD)	21.96 (1.98)	20.81 (1.53)	0.164
Female, No. (%)	7 (70)	8 (80)	1.000
**Clinical characteristics**^a^			
Antidepressant use, No. (%)	0 (0)	7 (77.78)	< 0.001*
BAI score, means (SD)	3.30 (3.68)	10.56 (14.70)	0.184
BDI score, means (SD)	6.55 (8.15)	16.60 (14.58)	0.081
PSS score, means (SD)	16.80 (6.47)	21.11 (7.21)	0.191

*HC* healthy controls, *MDD* major depressive disorder, *Age (Y)* Years of age; Body mass index (kg/m^2^), *BDI* Beck Depression Inventory, *BAI* Beck Anxiety Inventory, *PSS* Perceived Stress Scale.

^a^One MDD subject had missing data in antidepressant use and clinical scales.

### Differences in the gut microbiota composition and miRNAs between MDD patients and healthy controls

**Figure 1:**
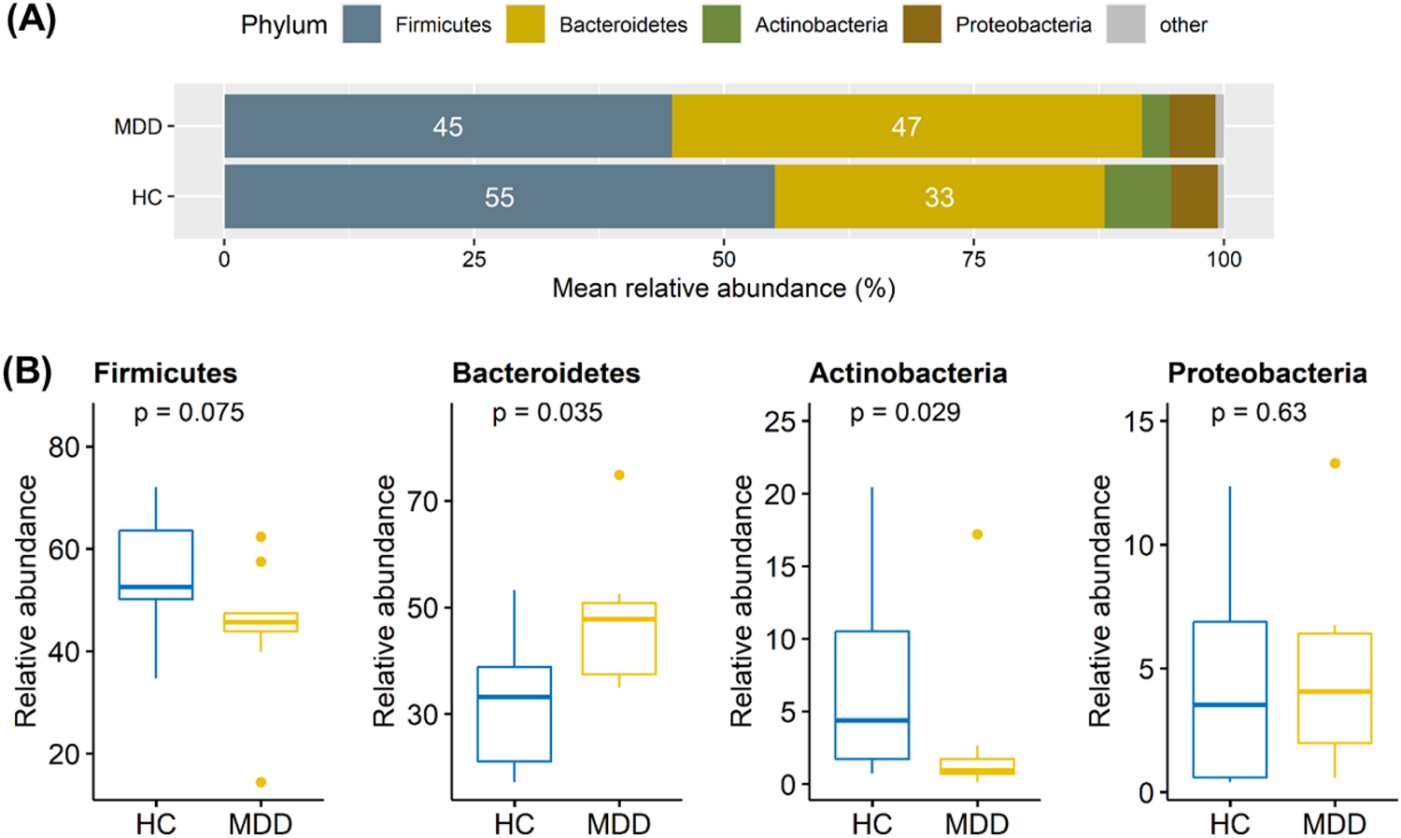
Microbiota composition at the phylum level. (**A**) Firmicutes and Bacteroidetes were the most dominant phyla within the groups, followed by Actinobacteria and Proteobacteria. (**B**) Two phyla Bacteroidetes and Actinobacteria significantly differed between the HC and MDD groups.

Firmicutes and Bacteroidetes dominated within almost all fecal samples, followed by Actinobacteria and Proteobacteria (Table S1 and Fig. 1A). Two phyla, Bacteroidetes and Actinobacteria, significantly differed between patients with MDD and healthy controls (Fig. 1B). At the genus level, a total of 270 genera were detected within the whole fecal samples. The alpha diversity, measured by richness and the Shannon diversity index, and the beta diversity, measured by the Bray–Curtis distance, did not show any marked differences between the two groups (Fig. S1).

**Figure 2:**
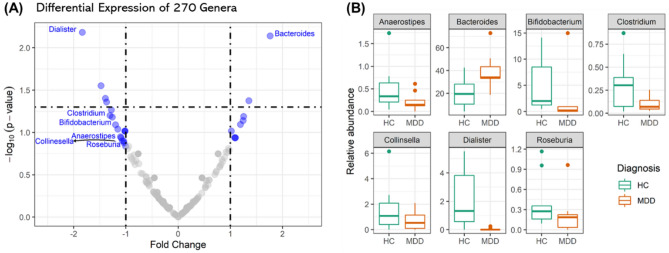
Volcan plot of 270 genera and boxplots of seven genera of interest. (**A**) The volcano plot displays the relationship between fold change (FC) and significance (p-value) between healthy controls and patients with MDD. The x-axis represents the difference in expression between two groups as log2 fold change; the y-axis represents the negative log10 of p-value using the Wilcoxson test. 27 blue dots indicate the absolute values of fold change greater than 1. (**B**) The boxplots depict the relative abundance of the seven genera in each group. These genera have absolute values of FC greater than one and were presented in over 90% of all fecal samples.

A total of 798 miRNAs were detected in the whole fecal samples. According to the results of the absolute values of fold changes (AFC) between healthy controls and patients with MDD, 27 genera (Tables S2 and Fig. 2A) and six miRNAs (Tables S3 and Fig. S2A) had AFC greater than one. Figure S2B depicts the expression values of the six miRNAs (miaR-579-3p, miR-1246, miR-1276, miR-1976, miR-4488, and miR-3144-3p) in patients with MDD and healthy controls. Three of the six miRNAs (miR-1276, miR-3144-3p, miR-1976) showed significant differences with p-values less than 0.05. Within 27 genera, only seven (*Anaerostipes*, *Bacteroides*, *Bifidobacterium*, *Clostridium*, *Collinsella*, *Dialister*, and *Roseburia*) were presented in over 90% of all fecal samples. Figure 2B depicts the distributions of the relative abundances of the seven genera in patients with MDD and healthy controls. We selected the seven genera to further analyze their relationships with 798 miRNAs.

### Candidate miRNAs that were significantly correlated with genera were highly associated with MDD

**Figure 3:**
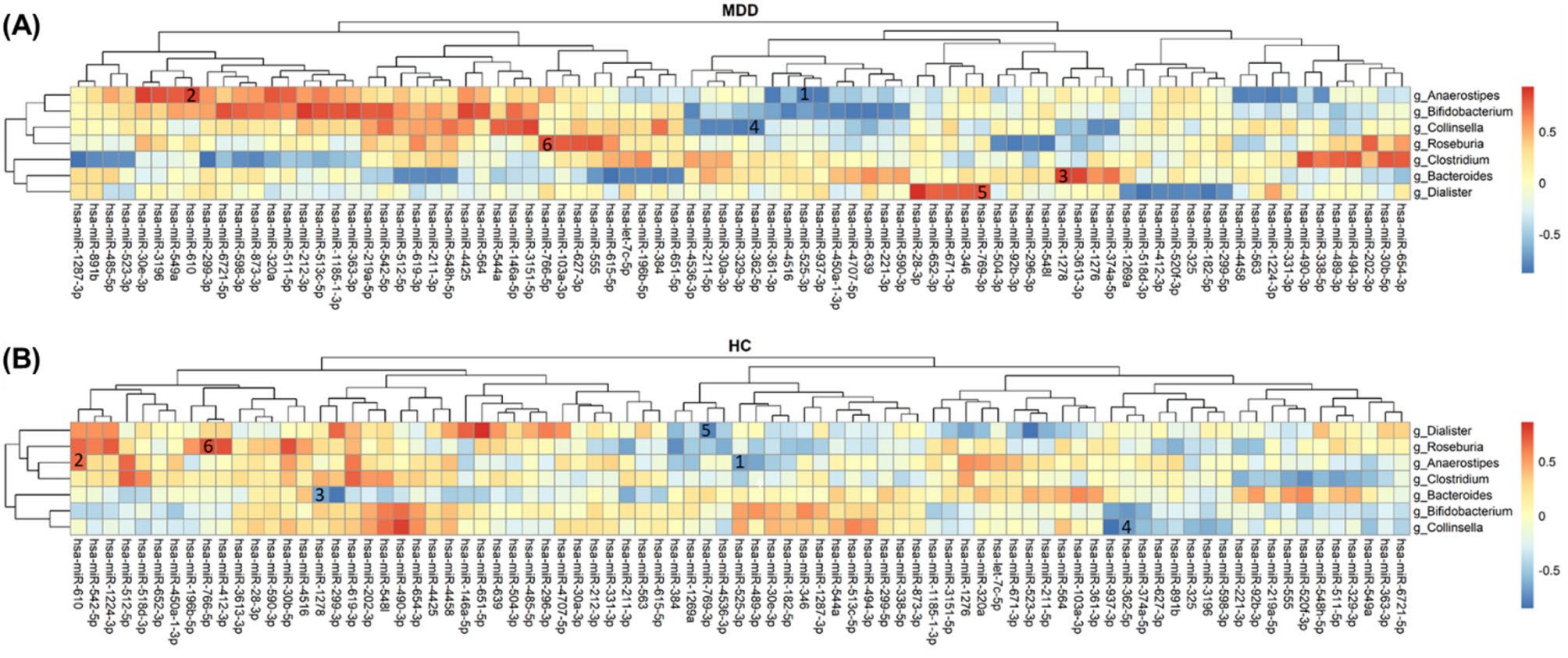
Two Correlation heatmaps between 7 genera and 83 miRNAs. The strength of correlation was measured by the Spearman correlation coefficient. Red indicates positive correlation, and blue indicates negative correlation. The numbers in some cells highlight the pairs which had significant correlation in both the HC and MDD groups. (**A**) In the MDD group, 36 positive-correlation pairs and 48 negative-correlation pairs had a p-value less than 0.01. (**B**) In the HC group, six pairs had significant correlations, which were (*Anaerostipes*, miR-525-3p), (*Anaerostipes*, miR-610), (*Bacteroides*, miR-1278), (*Collinsella*, miR-362-5p), *(Dialister*, miR-769-3p), and (*Roseburia*, miR-766-5p).

For the seven genera, we explored their correlation with the total of 798 miRNAs in patients with MDD. According to Spearman’s correlation coefficient results between the genera and the miRNAs, we found 48 negative-correlation pairs and 36 positive-correlation pairs, which had p-values less than 0.01 (Tables S4 and S5, Fig S3A). One miRNA (miR-598-3p) appeared in both sets with different genera (Fig S3B). The correlations between the seven genera and 83 miRNAs are shown in Fig. 3A. Within the genus-miRNA pairs in patients with MDD, the genus *Bifidobacterium* has the highest number of miRNAs with significant correlations (Fig. S3C). On the other hand, the correlations between the seven genera and 83 miRNAs within the healthy controls exhibited different patterns; only six pairs had significant correlations: (*Anaerostipes*, miR-525-3p), (*Anaerostipes*, miR-610), (*Bacteroides*, miR-1278), (*Collinsella*, miR-362-5p), (*Dialister*, miR-769-3p), and (*Roseburia*, miR-627-3p); as shown in Fig. 3B correlation heatmap. It is noted that in the healthy controls, *Bacteroides* and *Dialister* had strong negative correlations with miR-1278 and miR-769-3p, respectively, whereas both pairs had positive correlations in MDD patients (Tables S4 and S5).

Table S6 provides the overall correlation coefficients with p-values between the six miRNAs, which had AFC greater than one, and the seven genera in patients with MDD and healthy controls. Within these genus-miRNA pairs, twelve pairs had p-values less than 0.05 (Table S6). The seven genera had no significant correlation with miR-1246, whereas *Bifidobacterium* showed a positive correlation with miR-1976 in patients with MDD (r = 0.661, p-value = 0.0376) as well as in healthy controls (r = 0.709, p-value = 0.0217) (Table S6). Only *Collinsella* showed a strong negative correlation with miR-1276 (r = − 0.773, p-value = 0.0088) in patients with MDD (Tables S4 and S6).

### Predicted pathways of miRNAs associated with candidate genera

**Figure 4:**
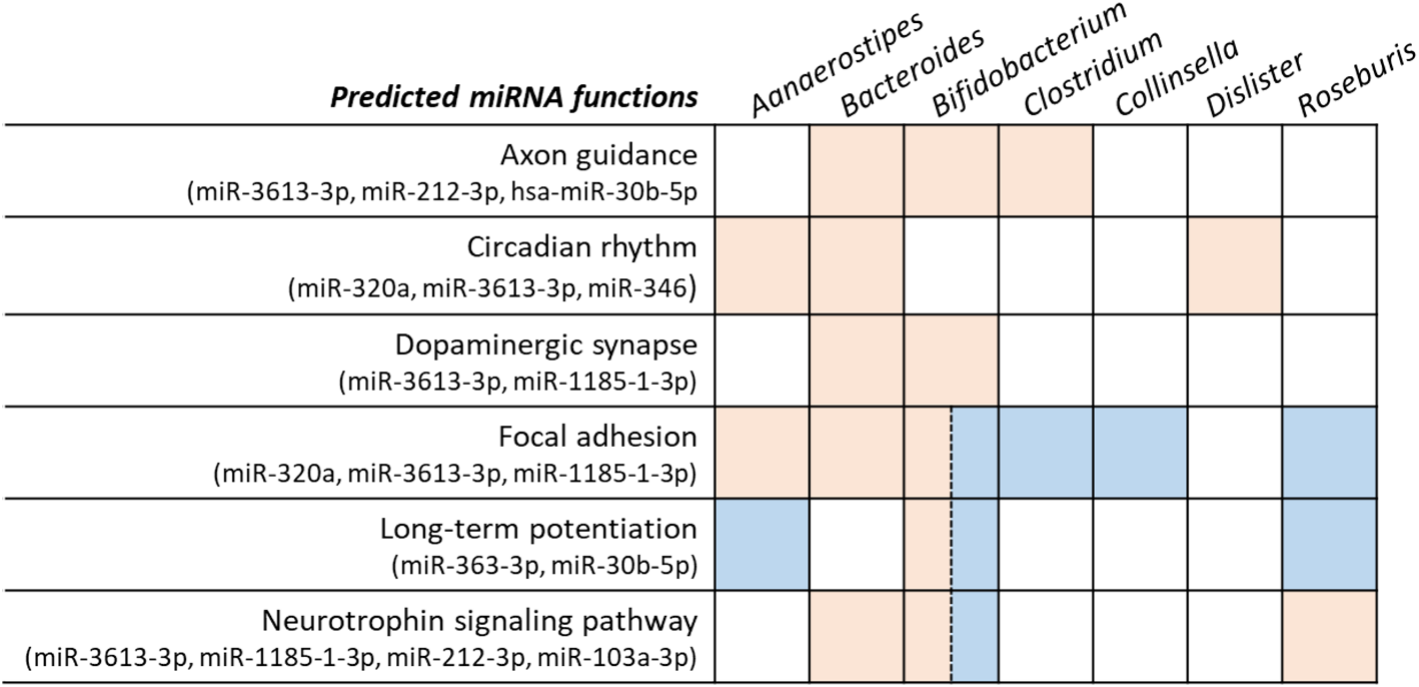
The relationship between six predicted miRNAs functions and seven genera of interest. The six predicted miRNA functions were constantly reported to be associated with major depressive disorder. The colored cells represent a strength of correlation with an absolute value greater than 0.5 and a p-value less than 0.01, between the genus and the miRNAs which regulate the pathway. Pink represents positive correlation, and blue represents negative correlation.

We derived predicted pathways of miRNAs separately, which had positive and negative correlations with the seven genera by DIANA-miRPath. Figures S4 and S5 are heatmaps showing the pathways for miRNAs function with a q-value of less than 0.001 (FDR corrected). Tables S7 and S8 depict the pathways and the correlations of the genera and miRNAs that regulated such pathways. Six pathways showed associations with MDD from previous studies, including axon guidance, circadian rhythm, dopaminergic synapse, focal adhesion, long-term potentiation, and neurotrophin signaling pathway. Tables 2 and 3 list these six pathways and the details of the involved miRNAs and genera, for which the pathways are frequently reported for MDD. It is noted that three pathways positively and negatively correlated with *Bifidobacterium.* This can be explained by *Bifidobacterium* showing positive correlation with miR-593-3p, which regulates focal adhesion pathway. Meanwhile, *Bifidobacterium* had a negative correlation with another miRNA (miR-1185-1-3p), which also regulates focal adhesion pathway. A similar result arises for long-term potentiation and neurotrophin signaling pathway, but with different miRNAs (Fig. 4).

**Table 2:** Three MDD-associated pathways which were regulated by specific miRNAs of interest. Six MDD-associated genera showed significantly negative correlations with these miRNAs.

Pathway		Genera with miRNAs		
	p-value	Genus	Correlation	p-value
**Focal adhesion (hsa04510)**				
hsa-miR-590-3p	9.52E−04	*Bifidobacterium*	− 0.770	0.0092
hsa-miR-891b	2.13E−04	*Clostridium*	− 0.782	0.0075
hsa-miR-374a-5p	9.84E−04	*Collinsella*	− 0.782	0.0075
hsa-miR-92b-3p	5.12E−05	*Roseburia*	− 0.833	0.0029
Union of miRNA target genes	9.44E−06			
**Long-term potentiation (hsa04720)**				
hsa-miR-338-5p	4.78E−05	*Anaerostipes*	− 0.766	0.0098
hsa-miR-590-3p	3.94E−06	*Bifidobacterium*	− 0.770	0.0092
hsa-miR-92b-3p	3.76E−04	*Roseburia*	− 0.833	0.0029
Union of miRNA target genes	9.44E−06			
**Neurotrophin signaling pathway (hsa04722)**				
hsa-miR-590-3p	9.64E−05	*Bifidobacterium*	− 0.770	0.0092
Union of miRNA target genes	9.44E−06

**Table 3:** Six MDD-associated pathways which were regulated by specific miRNAs of interest. Seven MDD-associated genera shoed significantly positive correlations with these miRNAs.

Pathway		Genera with miRNAs		
	p-value	Genus	Correlation	p-value
**Focal adhesion (hsa04510)**				
hsa-miR-320a	6.88E−06	*Aanaerostipes*	0.806	0.0049
hsa-miR-3613-3p	5.46E−06	*Bacteroides*	0.855	0.0016
hsa-miR-1185-1-3p	1.75E−04	*Bifidobacterium*	0.794	0.0061
Union of miRNA target genes	1.24E−09			
**Neurotrophin signaling pathway (hsa04722)**				
hsa-miR-3613-3p	1.18E−05	*Bacteroides*	0.855	0.0016
hsa-miR-1185-1-3p	3.61E−04	*Bifidobacterium*	0.794	0.0061
hsa-miR-212-3p	4.20E−04	*Bifidobacterium*	0.891	0.0005
hsa-miR-103a-3p	8.29E−04	*Roseburia*	0.784	0.0072
Union of miRNA target genes	1.95E−09			
**Axon guidance (hsa04360)**				
hsa-miR-3613-3p	3.04E−09	*Bacteroides*	0.855	0.0016
hsa-miR-212-3p	9.64E−04	*Bifidobacterium*	0.891	0.0005
hsa-miR-30b-5p	4.09E−08	*Clostridium*	0.794	0.0061
Union of miRNA target genes	2.74E−09			
**Circadian rhythm (hsa04710)**				
hsa-miR-320a	6.84E−06	*Aanaerostipes*	0.806	0.0049
hsa-miR-3613-3p	1.01E−10	*Bacteroides*	0.855	0.0016
hsa-miR-346	2.01E−06	*Dialister*	0.790	0.0065
Union of miRNA target genes	3.38E−09			
**Long-term potentiation (hsa04720)**				
hsa-miR-363-3p	4.23E−05	*Bifidobacterium*	0.848	0.002
hsa-miR-30b-5p	2.02E−04	*Clostridium*	0.794	0.0061
Union of miRNA target genes	5.53E−05			
**Dopaminergic synapse (hsa04728)**				
hsa-miR-3613-3p	3.90E−04	*Bacteroides*	0.855	0.0016
hsa-miR-1185-1-3p	3.60E−04	*Bifidobacterium*	0.794	0.0061
Union of miRNA target genes	5.54E−04

## Discussion

The current pilot study explored the relationships between host miRNAs and gut microbiota obtained from fecal samples for depression. Using bioinformatics analysis, our results demonstrated that the relative abundance of seven genera significantly differed between the healthy controls and patients with MDD. The seven genera are also highly correlated with some miRNAs. According to the results of the predicted miRNA functional profiles, these miRNAs seemed to potentially be involved in regulating specific pathways for depression.

Approximately 80 or more distinct circulating miRNAs were reported to be associated with depression in human studies in a systematic review^28^. For example, using blood or brain samples, several studies on mental disorder-related traits have suggested the biological roles of let-7b, let-7c, miR-124-3p, and miR-323a influence stress response and neural plasticity^29–31^. Three miRNAs, miR-132, miR-17-5p, and let-7a-5p, were reported in more than one study in MDD patients^24,28^. All these findings indicate the potential involvement of circulating miRNAs’ in depression. By contrast, no fecal miRNAs have been investigated in MDD-related studies. In the current study, six fecal miRNAs (miR-1246, miR-579-3p, miR-1276, miR-1976, miR-3144-3p, miR-4488) showed differential expressions between patients with MDD and healthy controls, while three of them reached statistical significance. None of these miRNAs were ever reported in previous studies using blood or tissue samples^16^. A recent study revealed that plasma and serum miRNAs strongly correlate with adipose, liver, and spleen miRNAs, whereas fecal miRNAs are highly associated with miRNAs from the gastrointestinal tract, such as the stomach, the small intestine, and the colon^4,32^. Thus, one of the reasons for the inconsistent findings could be due to different biosample types.

The gut microbiome plays a vital role in brain physiology, psychology, and behaviour. The seven genera in our pilot study significantly differed between patients with MDD and healthy controls. For instance, the two genera *Bifidobacterium* and *Dialister* were markedly less abundant in MDD patients than in healthy controls, which is consistent with the findings from the previous studies^18,34,35^. In contrast, patients with MDD had a higher relative abundance of the phylum Bacteroidetes and the genus *Bacteroides* compared to the healthy controls. Using mice models, the microbial colonization process was found to modulate depressive and anxious behaviours^36^. Nevertheless, according to a systematic review and meta-analysis of existing human studies in depression, no consistent targeted microbiota was specified for MDD^16,37^. The inconsistent findings are possibly due to ethnical differences, uncontrolled confounding factors, and small sample size in general. It remains unclear as to how the microbiota is naturally regulated in the human host, which is related to abnormal emotional regulation.

In our pilot study, the three significant fecal miRNAs, which had AFC greater than one between the healthy controls and patients with MDD, were correlated with some of the genera of interest (Tables S4-S6), and also participated in specific predicted biological pathways (Table S7). Fecal miRNAs could affect the MDD-associated biological pathways through different ways, and the gut microbiome can be shaped by miRNAs^25,26,38,39^. For instance, studies in colon cancer have shown that patients with high amounts of tissue *Fusobacterium nucleatum* and miR-21 were at a higher risk of poor outcomes^39^. Additionally, a recent animal study provided evidence for a potential mechanism for the interaction of host miRNA-microbe in gastrointestinal homeostasis^40^. Therefore, miRNAs which were strongly associated with the seven genera of interest could be involved in the microbial colonization process, as well as the MDD-associated biological systems directly or indirectly.

Microbiota-gut-brain axis signaling has been recognized as pivotal in mood regulation, such as anxiety and depression^33^. The immune, endocrine, and nervous systems are the main systems to provide pathways between the human gut and the brain^12^. The enteric microenvironment responds to microbial factors, mainly via pattern recognition receptors such as Toll-like receptors, neurotransmitters, neuropeptides, and neurohormone receptors^41–43^. In view of the biological pathways for MDD, axon guidance, circadian rhythm, dopaminergic synapse, long-term potentiation, and neurotrophin signaling pathway are frequently reported to be associated with MDD^44–47^. In this study, using bioinformatics analysis, we found that these pathways were also regulated by the fecal miRNAs that are highly correlated with specific genera identified in the current study. Microbial metabolites are able to enter the bloodstream and travel around in the host body and further affect brain disorder directly or indirectly. A number of routes have been proposed with a certain level of evidence for the communication between gut microbiota and brain phenotypes^48^.The communication between the host and the gut microbiota is a complex regulatory network. Overall, these findings offer clues to bridge the gaps in existing knowledge related to the link between gut microbiota and the brain, though further studies are required to elucidate the causal relationship and underlying mechanisms between the two.

The current pilot study explores the relationships between fecal miRNAs and gut genera in patients with MDD using bioinformatics analysis. Nevertheless, our findings by no means determine the fundamental biological mechanism behind such associations. This pilot study has some limitations. Firstly, due to the limited number of samples, we only considered analyses for the miRNAs that are highly correlated with the seven genera identified in the present study without rigorous p-value adjustment of multiple testing. Our results require to be validated using a larger sample size. Secondly, details of medication treatment and lifestyle factors, which potentially affect gut microbiota composition, were not thoroughly explored. Further studies should include detailed medication records, diet, exercise, etc. Thirdly, 16S rRNA gene amplicon sequencing generates relative abundance of taxa or genes within the microbial community. It provides neither absolute abundance information nor specific bacteria data at the species and strain levels. In addition, this pilot study used two primer designs and DNA extraction protocols. We cannot rule out the possibility that these may bias results to some degree from the sample processing pipeline and the batch effects.

In the future, metagenomic shotgun sequencing will provide more informative results than the 16S rRNA amplicon sequencing employed in this study. Shotgun sequencing data could reveal the functions of microbial communities, as well as the interactions with the host factors across a larger sample size. In order to validate any suggesting links between possible miRNA-correlated systemic effects and brain phenotypes, further studies would be needed, such as other model systems, to assist causal inference in this topic and to provide more clues for specific microbes and miRNA candidates in MDD-associated biological systems of interest.

## Materials and methods

### Study participants

The diagnosis of MDD was based on the criteria of the Diagnostic and Statistical Manual of Mental Disorders fifth Edition (DSM-5)^49^. Patients with MDD diagnosis were referred to the study by psychiatrists from several central and regional hospitals in Taipei. Patients diagnosed with schizophrenia, schizoaffective disorder, intellectual disability, or substance-induced secondary MDD were excluded from the study. Control participants with no history of major psychiatric disorders were collected from the local community in the same catchment area of cases. Participants were excluded if they had regularly taken antibiotics, probiotics, prebiotics, or symbiotics, been diagnosed with active bacterial, fungal, or viral infections, or had undergone gastrointestinal surgery in the past two months prior to sample collection.

Participants of the present study were drawn from the Genomic Research and Epidemiological Studies for Affective Disorders in Taiwan, for which subjects provided complete phenotypic and lab data of 16S rRNA gene amplification sequences and fecal miRNA analysis were included. In total, there consisted of 10 healthy controls and 10 MDD patients. Approval of this study was obtained from the institutional review board of participating hospitals (*e.g.*, project number 201512086RIND). Written informed consent was obtained from every participant after the study procedures had been fully explained.

### Beck Depression Inventory, Beck Anxiety Inventory, and Perceived Stress Scale

The level of symptom severity was measured using Beck Depression Inventory (BDI) which assessed current depressive severity within two weeks. The cutoff of 13, 19, and 28 were set to indicate minimal, mild, moderate, and severe depression. The anxiety level among individuals was measured using Beck Anxiety Inventory (BAI) within one week. A score cutoff of 9, 16 and 29 was used to indicate minimal, mild, moderate, and severe anxiety. The stress level was measured using Perceived Stress Scale (PSS). Scores of 0–13, 14–26, or 27–40 represent low, moderate, and high perceived stress, respectively.

### Fecal sample collection and 16S rRNA gene amplification sequences

Fecal samples were self-collected by participants at home and were put in the refrigerator before delivering to the laboratory the next day. Approximately 2 g of the fecal samples were collected, and all fecal samples were delivered at 4 °C and stored in a − 80 °C refrigerator immediately after transferring to 2 cc eppendorf. Bacterial DNA was isolated from fecal samples using the bead-beating method as previously described^15^. Amplicon sequencing reads were generated from two types of amplicon primer, V4 region and V3-V4 region, in two different sequencers. The V4 region of the 16S rRNS gene was amplified from 8 extracted DNA samples, and then sequenced using the Illumina MiniSeq 2 × 250 bp platform (V4 region: 515F- TGCCAGCMGCCGCGGTAA and 806R-GGACTACNNGGGTATCTAAT primer). The V3-V4 region of the 16S rRNA genes (V3-V4) was amplified from 12 extracted DNA samples, and then sequenced using the Illumina MiSeq 2 × 300 bp platform (V3-V4 region: Forward Primer CCTACGGGNGGCWGCAG and Reverse Primer GACTACHVGGGTATCTAATCC).

### Quality control and operational taxonomic units

The total number of raw sequencing reads was 9,407,211 in 8 fecal samples using MiniSeq, and 3,264,475 in 12 fecal samples using MiSeq. The low-quality raw sequencing reads were filtered using “fastq_quality_filter” with parameters “-q 20 -p 70”. Then the adapters and primers were trimmed using “fastx_clipper. Two types of amplicon sequencing reads were merged using “pandaseq.” The merged sequences were further reversed and complemented for the V3-V4 regions of 16S rRNA gene sequences. The V3 region was then removed with the reverse complement primer of 515F (TTACCGCGGCNGCTGGCAC) using “fastx_clipper.” Finally, the reverse complement of the remaining sequences was the V4 region of the 16S rRNA gene. Operational taxonomic units (OTUs) were picked using the closed-reference picking script in QIIME v1.7.0 and the Greengenes database version 13.5 with 97% similarity^7,50^. Based on the 20 fecal samples, 15,392,451 sequencing reads were aligned, identifying an average of 40,937 sequencing reads per sample. We sorted the sequences into 548 operational taxonomic units (OTUs, ≥ 97% ID).

### Fecal miRNA isolation and analysis

The extraction of total RNA from feces was based on the method described by Reck et al.^51^, and we used commercially available kits to isolate total RNA from small amounts of stool samples (PowerMicrobiome™ RNA Isolation Kit, MoBio Laboratories, Germany). In short, approximately 0.25 g of feces was transferred to a glass bead tube (0.1 mm). Shortly before the feces sample was completely thawed on ice, added β-mercaptoethanol to the MoBio lysis buffer to make the final concentration of 10 μl/ml in the tube, and homogenized on the Vortex-Genie 2 Mixer at the highest speed for 10 min. The sample was centrifuge at 13,000×*g* for 1 min at room temperature, and further steps included DNAse I treatment of the samples and elution of RNA in nuclease-free water, which were performed according to the manufacturer's instructions. The RNA concentration was measured using Nanodrop ND 2000 (Thermo Scientific, USA).

The miRNA profiles in human feces were determined by NanoString nCounter System (NanoString Technologies) and the nCounter Human-v2 miRNA Panel with ~ 800 unique miRNA barcodes. Assays and spike-in controls were used for normalization based on identical amounts of input RNA.

### Statistical analysis

Differences in the relative abundance of phyla and genera, alpha diversity, and beta diversity were evaluated by the Wilcoxon rank sum test. The principal coordinate analysis method was used for the visualization of beta diversity. Log-transformation with base 2 and quantile normalization was used for miRNAs expression levels. Differential miRNA expression between MDD patients and healthy controls was visualized by a volcano plot. The correlation analysis between the candidate genera and miRNAs was measured by the Spearman’s rank correlation coefficient, and the correlation matrix was generated by the rcorr() function package in R. Significant correlations between genera and miRNAs were defined as the absolute values of correlation coefficients of over 0.5, with a p-value less than 0.05. Heatmaps of the correlations were generated in R. Student’s-*t* test was used to compare variables between groups, including age, body mass index, and clinical scales. The predicted miRNA pathways were generated by DIANA-miRPath from the DIANA-microT-CDS algorithm and KEGG^52^. The p-value threshold for reporting signals was set to 0.001 and MicorT threshold to 0.8, and multiple comparisons were corrected using a false discovery rate < 0.05. R software was used for all statistical analyses (The R Project for Statistical Computing, Vienna, Austria).

### Ethical statement

The authors assert that all procedures contributing to this work comply with the ethical standards of the Helsinki Declaration of 1975, as revised in 2008. Approval of this study was obtained from the Research Ethics Committee, National Taiwan University Hospital (201512086RIND).

## Supplementary Information

Supplementary Information.

## Data Availability

The raw sequence data from the 16S rRNA gene amplicon sequencing experiments are deposited in the NCBI Sequence Read Archive under Bio-Project accession number PRJNA890246. https://dataview.ncbi.nlm.nih.gov/object/PRJNA890246?reviewer=2090md2ma29thudhf9b3lhjeue.
