# Supplementary material for: Exploration of the relationship between gut microbiota and fecal microRNAs in patients with major depressive disorder
Source: Sci Rep. 2022 Dec 5;12:20977. doi: 10.1038/s41598-022-24773-7 (PMC9722658; doi:10.1038/s41598-022-24773-7)
Supplement: Supplementary file 1 — Supplementary Information. [file 41598_2022_24773_MOESM1_ESM.pdf]

Supplemental Information

Exploration of the relationship between gut microbiota and fecal microRNAs in patients with major depressive disorder

Hui-Mei Chen, Yu-Chu Ella Chung, Hsi-Chung Chen, Yen-Wenn Liu, I-Ming Chen, Mong-Liang Lu, Felix Shih-Hsiang Hsiao, Chun-Hsin Chen, Ming-Chyi Huang, Wei-Liang Shih, Po-Hsiu Kuo

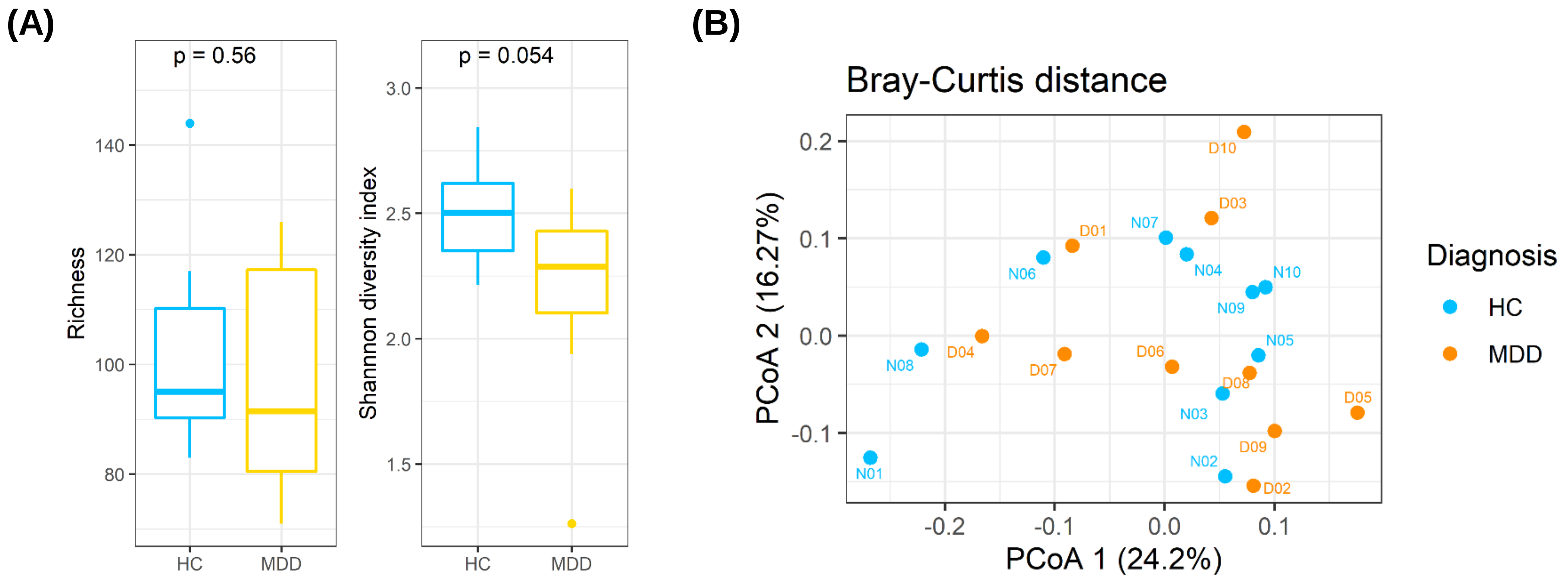

Figure S1. Microbial diversity at the genus level. (A) Alpha diversity was assessed using richness and the Shannon diversity index. The richness of microbial diversity means the number of genera detected within each fecal sample. Both richness and the Shannon diversity index did not show significant differences between the HC and MDD groups. (B) Principal coordinate analysis (PCoA) was based on the Bray-Curtis distance**.** Each point represents one fecal sample, with blue denoting the HC group, and orange denoting the MDD group.

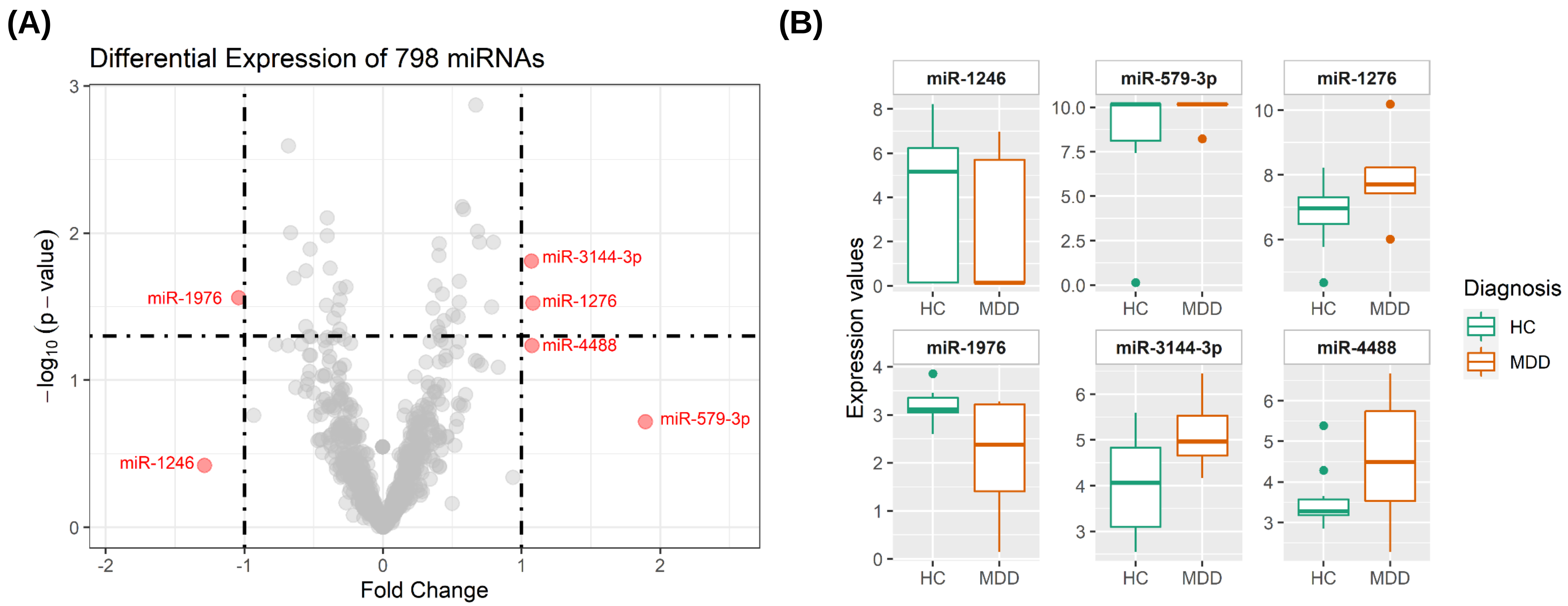

Figure S2. Volcano plot of 798 miRNAs and boxplots of six miRNAs. (A) The volcano plot displays the relationship between fold change and significance (p-value) between healthy controls and patients with MDD. The x-axis represents the difference in expression between two groups as log2 fold change; the y-axis represents the negative log10 of p-value using the Wilcoxson test. Six red dots indicate absolute values of fold change (AFC) greater than 1. **(B)** The boxplots depict the relative abundance of the six miRNAs with AFC greater than 1 in each group.

(A)

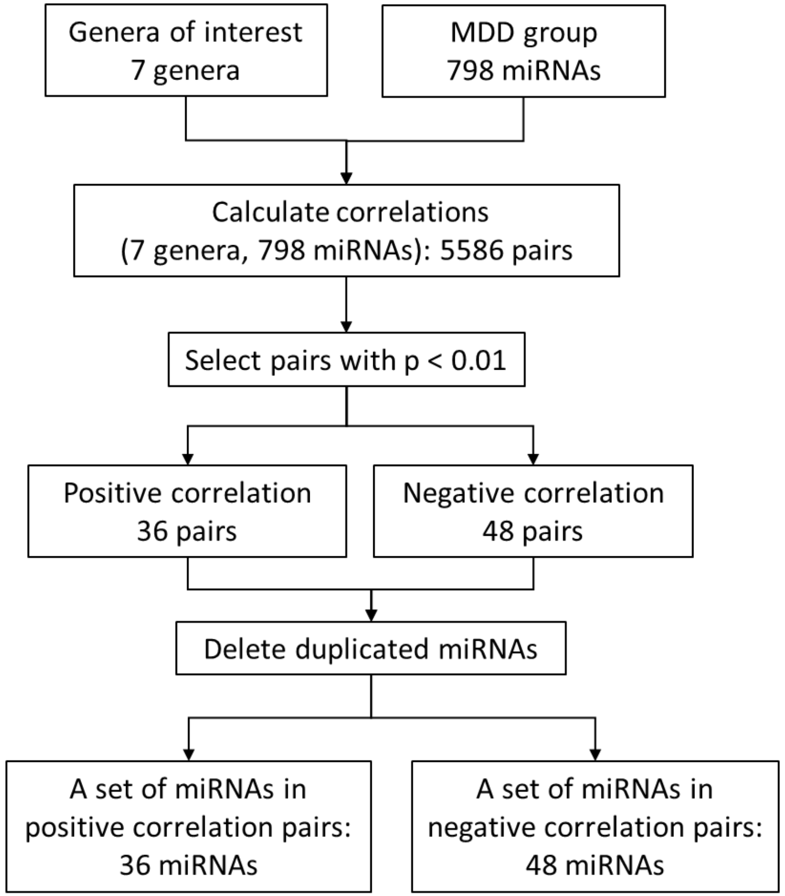

(B)

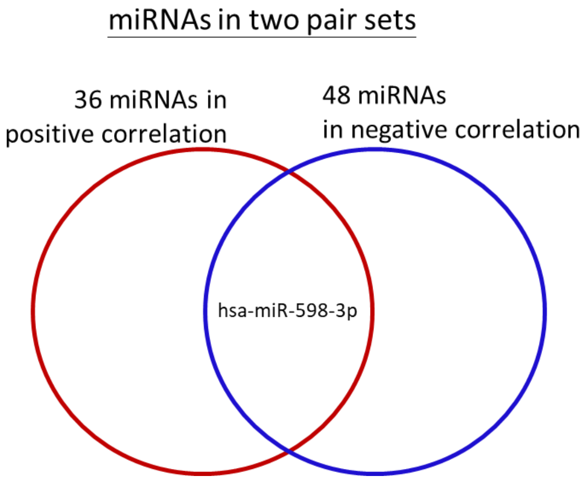

(C)

Genus	**Number of miRNAs**	
	positive	negative
Anaerostipes	6	8
Bacteroides	2	9
Bifidobacterium	10	7
Clostridium	5	7
Collinsella	3	6
Disalister	5	7
Roseburia	5	4
Total	36	48

Figure S3. (A) An overview of analysis processes to select miRNAs which had significant correlation with genera of interested. The correlations between genera and miRNAs were measured by Spearman's correlation coefficient. (B) A Venn diagram of two correlation sets. The red circle represents 36 positive-correlation pairs, and the blue circle represents 48 negative-correlation pairs. These pairs had a p-value less than 0.01. (C) A simplified table of the number of miRNAs which positively and negatively associated with each genus of interest in patients with MDD.

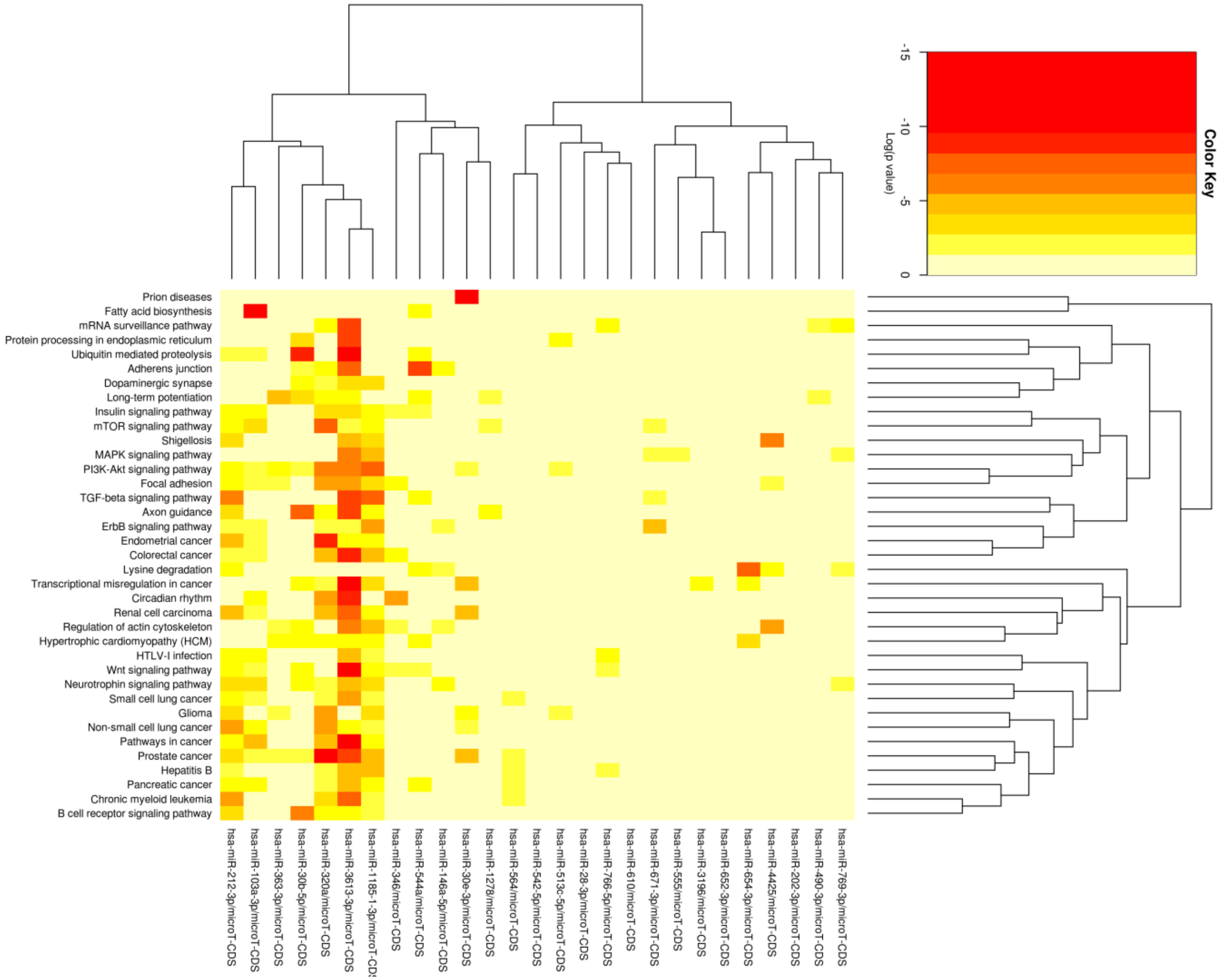

Figure S4. Heatmap showing top significant predicted miRNA pathways in 36 miRNAs. Of the 36 miRNAs which had significant, positive correlation with the seven genera (*Anaerostipes*, *Bacteroides*, *Bifidobacterium*, *Clostridium*, *Collinsella*, *Dialister*, and *Roseburia*), 9 miRNAs were excluded from the pathway analysis as they were not included in the database. After FDR correction the results were again filtered to include only pathways with $p < 0.001$. Fisher's Exact test (hypergeometeric distribution) was used for statistical analysis. Consequently, 37 predicted miRNA pathways and 27 miRNAs were shown in the heatmap.

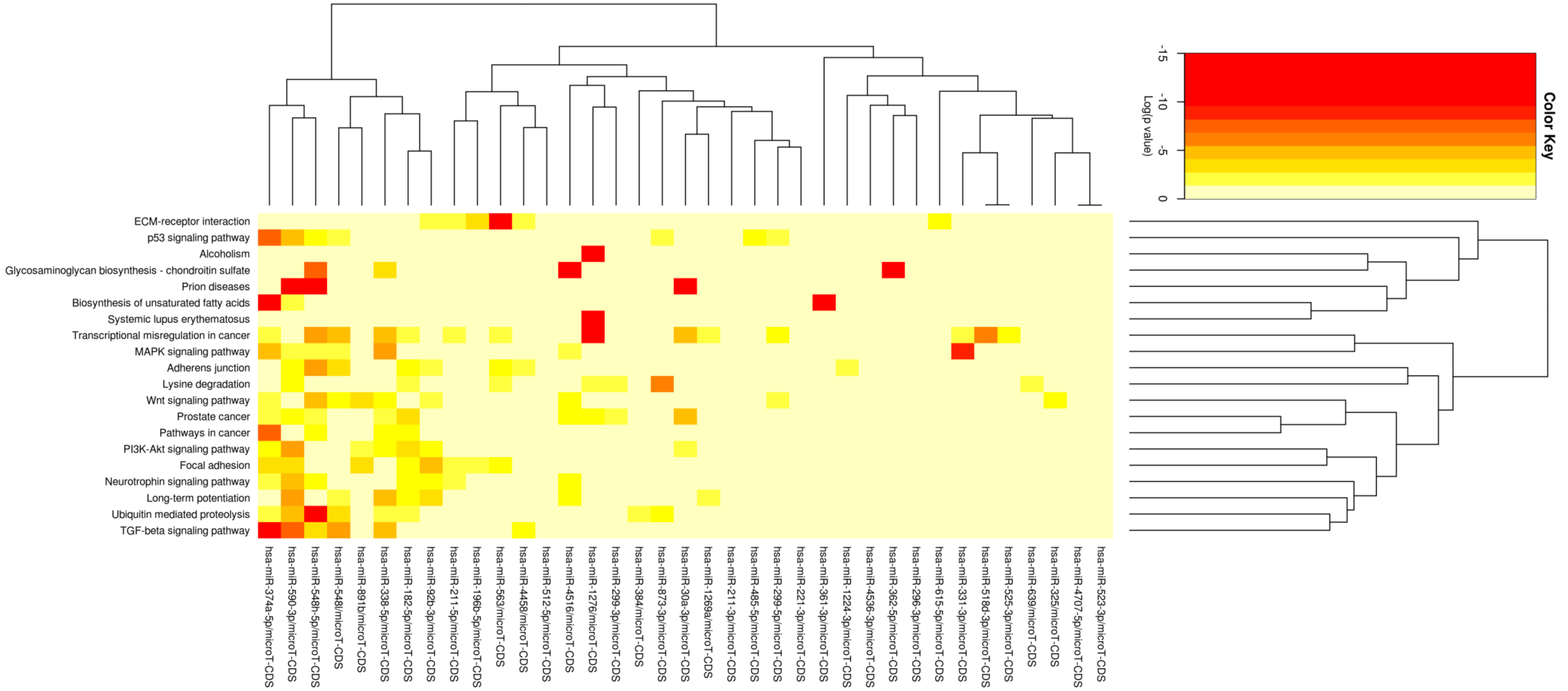

Figure S5. Heatmap showing top significant predicted miRNA pathways in 45 miRNAs. Of the 45 miRNAs which had significant, negative correlation with the seven genera (*Anaerostipes*, *Bacteroides*, *Bifidobacterium*, *Clostridium*, *Collinsella*, *Dialister*, and *Roseburia*), 11 miRNAs were excluded from the pathway analysis as they did were not included in the database. After FDR correction the results were again filtered to include only pathways with p <0.001. Fisher's Exact test (hypergeometeric distribution) was used for statistical analysis. Consequently, 20 predicted miRNA pathways and 36 miRNAs were shown in the heatmap.

Tabel S1. Mean realtive abundance and percentange of detected fecal samples

	Mean relative abundace		Percentage of detected samples		
Phylum	HC	MDD	in the HC group	in the MDD group	p-value
Firmicutes	55.0543178	44.799144	100	100	0.0753
Bacteroidetes	33.0038323	47.001951	100	100	0.0355
Actinobacteria	6.6527096	2.708903	100	100	0.0288
Proteobacteria	4.6439646	4.662602	100	100	0.6305
Fusobacteria	0.4205819	0.693382	90	80	0.7910
Tenericutes	0.0706650	0.072766	30	40	0.6898
Verrucomicrobia	0.1140983	0.020458	30	60	0.4318
Lentisphaerae	0.0308961	0.007190	30	20	0.7281
Cyanobacteria	0.0017821	0.016323	100	100	0.2475
Synergistetes	0.0030251	0.008589	70	60	0.9692
TM7	0.0040873	0.004262	100	80	0.4725
Euryarchaeota	0.0000000	0.004309	0	10	0.3681
Spirochaetes	0.0000000	0.000085	0	10	0.3681
Planctomycetes	0.0000399	0.000000	10	0	0.3681
Chloroflexi	0.0000000	0.000037	0	10	0.3681

Table S2. Genera with the absolute values of fold change greater than 1

Genus Level	FC	absFC	p-value	log (p-value)	Percentage of fecal samples (N/20 %)
g_Dialister	-1.8360	1.8360	0.0066	5.025	90
g_Bacteroides	1.7625	1.7625	0.0072	4.930	100
g_Paraprevotella	-1.4767	1.4767	0.0280	3.575	55
g_Parvimonas	-1.3933	1.3933	0.0396	3.230	40
g_Enterococcus	-1.3713	1.3713	0.0437	3.130	60
g_Nelumbo	1.3545	1.3545	0.0422	3.165	30
g_Clostridium	-1.3059	1.3059	0.0630	2.764	100
g_Peptoniphilus	-1.2790	1.2790	0.0542	2.915	30
f_Oxalobacteraceae_uncharacterized	-1.2595	1.2595	0.0660	2.718	85
g_[Prevotella]	1.2548	1.2548	0.0648	2.737	70
g_Anaerotruncus	1.2442	1.2442	0.0723	2.628	75
g_Bifidobacterium	-1.1997	1.1997	0.0813	2.510	100
g_Bradyrhizobium	1.1929	1.1929	0.0805	2.520	45
f_.Barnesiellaceae._uncharacterized	-1.1559	1.1559	0.0914	2.392	60
g_Coprobacillus	-1.1058	1.1058	0.1127	2.183	65
g_Megamonas	1.0969	1.0969	0.1153	2.160	75
g_Desulfovibrio	1.0931	1.0931	0.1160	2.154	65
g_Anaerostipes	-1.0928	1.0928	0.1157	2.157	100
g_Acidaminococcus	1.0806	1.0806	0.1150	2.163	75
g_Collinsella	-1.0685	1.0685	0.1257	2.074	95
g_Mitsuokella	-1.0408	1.0408	0.1270	2.063	50
f_Rikenellaceae_uncharacterized	-1.0359	1.0359	0.1336	2.013	100
o_SHA.98_uncharacterized	1.0183	1.0183	0.0963	2.340	15
f_Pseudomonadaceae_uncharacterized	-1.0183	1.0183	0.0963	2.340	15
g_Proteus	-1.0183	1.0183	0.0963	2.340	15
g_Synechococcus	-1.0183	1.0183	0.0963	2.340	15
g_Roseburia	-1.0067	1.0067	0.1457	1.927	100

Table S3. miRNAs with the absolute values of fold change greater than 1

miRNAs	mean.HC	mean.MDD	sd.HC	sd.MDD	FC	Abs(FC)	p-value
hsa-miR-579-3p	7.9028	9.7956	4.179	0.826	1.8928	1.8928	0.1912
hsa-miR-1246	3.8249	2.5355	3.282	3.110	-1.2894	1.2894	0.3791
hsa-miR-1276	6.7761	7.8575	1.004	1.045	1.0814	1.0814	0.0298
hsa-miR-4488	3.5533	4.6272	0.752	1.457	1.0738	1.0738	0.0580
hsa-miR-3144-3p	4.0304	5.1003	1.047	0.668	1.0699	1.0699	0.0155
hsa-miR-1976	3.1703	2.1281	0.346	1.238	-1.0422	1.0422	0.0274

Table S4. Strong negative correlatoins between seven genera and miRNAs in the MDD group

Genus	NO	miRNA	MDD_cor	HC_cor	MDD.p-value	HC.p-Value
g_Anaerostipes	1	hsa-miR-1224-3p	-0.830	0.267	0.0029	0.4550
	2	hsa-miR-331-3p	-0.772	0.321	0.0089	0.3655
	3	hsa-miR-338-5p	-0.766	-0.091	0.0098	0.8028
	4	hsa-miR-361-3p	-0.842	0.006	0.0022	0.9867
	5	hsa-miR-4458	-0.818	0.394	0.0038	0.2600
	6	hsa-miR-525-3p	-0.833	-0.683	0.0028	0.0296
	7	hsa-miR-563	-0.794	-0.273	0.0061	0.4458
	8	hsa-miR-937-3p	-0.830	0.067	0.0029	0.8548
g_Bacteroides	1	hsa-let-7c-5p	-0.796	0.273	0.0058	0.4458
	2	hsa-miR-196b-5p	-0.799	-0.049	0.0056	0.8939
	3	hsa-miR-211-3p	-0.806	-0.612	0.0049	0.0600
	4	hsa-miR-384	-0.830	0.018	0.0029	0.9602
	5	hsa-miR-512-5p	-0.772	-0.442	0.0089	0.2004
	6	hsa-miR-548h-5p	-0.766	0.176	0.0098	0.6272
	7	hsa-miR-615-5p	-0.869	-0.479	0.0011	0.1615
	8	hsa-miR-619-3p	-0.784	-0.358	0.0072	0.3104
	9	hsa-miR-651-5p	-0.806	-0.468	0.0049	0.1725
g_Bifidobacterium	1	hsa-miR-221-3p	-0.815	-0.139	0.0041	0.7009
	2	hsa-miR-450a-1-3p	-0.806	-0.505	0.0049	0.1369
	3	hsa-miR-4516	-0.809	-0.030	0.0046	0.9338
	4	hsa-miR-4536-3p	-0.802	-0.134	0.0052	0.7118
	5	hsa-miR-4707-5p	-0.842	-0.042	0.0022	0.9074
	6	hsa-miR-590-3p	-0.770	0.285	0.0092	0.4250
	7	hsa-miR-639	-0.830	-0.018	0.0029	0.9602
g_Clostridium	1	hsa-miR-1287-3p	-0.879	0.128	0.0008	0.7253
	2	hsa-miR-299-3p	-0.867	0.383	0.0012	0.2747
	3	hsa-miR-485-5p	-0.770	0.055	0.0092	0.8810
	4	hsa-miR-523-3p	-0.770	0.006	0.0092	0.9867
	5	hsa-miR-598-3p	-0.770	0.055	0.0092	0.8807
	6	hsa-miR-873-3p	-0.796	-0.297	0.0058	0.4047
	7	hsa-miR-891b	-0.782	0.091	0.0075	0.8028
g_Collinsella	1	hsa-miR-1276	-0.773	-0.276	0.0088	0.4400
	2	hsa-miR-211-5p	-0.794	-0.207	0.0061	0.5667
	3	hsa-miR-30a-3p	-0.794	0.248	0.0061	0.4888
	4	hsa-miR-329-3p	-0.830	-0.127	0.0029	0.7261
	5	hsa-miR-362-5p	-0.818	**-0.681**	0.0038	0.0302
	6	hsa-miR-374a-5p	-0.782	-0.552	0.0075	0.0984
g_Dialister	1	hsa-miR-1269a	-0.768	-0.358	0.0094	0.3104
	2	hsa-miR-182-5p	-0.857	-0.370	0.0015	0.2931
	3	hsa-miR-299-5p	-0.766	-0.236	0.0098	0.5109
	4	hsa-miR-325	-0.772	-0.188	0.0089	0.6032
	5	hsa-miR-412-3p	-0.790	0.321	0.0065	0.3655
	6	hsa-miR-518d-3p	-0.863	0.261	0.0013	0.4671
	7	hsa-miR-520f-3p	-0.802	-0.285	0.0052	0.4250
g_Roseburia	1	hsa-miR-296-3p	-0.772	0.055	0.0089	0.8807
	2	hsa-miR-504-3p	-0.782	0.176	0.0075	0.6272
	3	hsa-miR-548l	-0.830	0.297	0.0029	0.4047
	4	hsa-miR-92b-3p	-0.833	-0.539	0.0028	0.1076

Table S5. Strong positive correlations between seven genera and miRNAs in the MDD group

Genus	NO	miRNA	MDD_cor	HC_cor	MDD.p-value	HC.p-Value
g_Anaerostipes	1	hsa-miR-30e-3p	0.881	-0.491	0.0007	0.1497
	2	hsa-miR-3196	0.818	-0.030	0.0038	0.9338
	3	hsa-miR-320a	0.806	0.535	0.0049	0.1111
	4	hsa-miR-511-5p	0.766	-0.292	0.0098	0.4133
	5	hsa-miR-549a	0.790	-0.462	0.0065	0.1789
	6	hsa-miR-610	0.867	0.661	0.0012	0.0376
g_Bacteroides	1	hsa-miR-1278	0.874	-0.661	0.0009	0.0376
	2	hsa-miR-3613-3p	0.855	-0.224	0.0016	0.5334
g_Bifidobacterium	1	hsa-miR-1185-1-3p	0.794	-0.479	0.0061	0.1615
	2	hsa-miR-212-3p	0.891	0.297	0.0005	0.4047
	3	hsa-miR-219a-5p	0.782	-0.413	0.0075	0.2351
	4	hsa-miR-363-3p	0.848	-0.268	0.002	0.4536
	5	hsa-miR-4425	0.855	0.285	0.0016	0.4250
	6	hsa-miR-513c-5p	0.816	0.309	0.004	0.3848
	7	hsa-miR-542-5p	0.794	-0.430	0.0061	0.2145
	8	hsa-miR-564	0.806	0.000	0.0049	1.0000
	9	hsa-miR-598-3p	0.770	-0.128	0.0092	0.7253
	10	hsa-miR-6721-5p	0.828	-0.375	0.0031	0.2850
g_Clostridium	1	hsa-miR-30b-5p	0.794	0.406	0.0061	0.2443
	2	hsa-miR-489-3p	0.770	-0.152	0.0092	0.6761
	3	hsa-miR-490-3p	0.833	0.309	0.0028	0.3848
	4	hsa-miR-494-3p	0.804	-0.329	0.0051	0.3528
	5	hsa-miR-654-3p	0.782	0.333	0.0075	0.3466
g_Collinsella	1	hsa-miR-146a-5p	0.782	-0.030	0.0075	0.9338
	2	hsa-miR-3151-5p	0.855	-0.321	0.0016	0.3655
	3	hsa-miR-544a	0.766	0.418	0.0098	0.2291
g_Dialister	1	hsa-miR-28-3p	0.939	-0.134	0.0001	0.7126
	2	hsa-miR-346	0.790	-0.299	0.0065	0.4017
	3	hsa-miR-652-3p	0.768	0.055	0.0094	0.8810
	4	hsa-miR-671-3p	0.790	-0.511	0.0065	0.1315
	5	hsa-miR-769-3p	0.809	-0.782	0.0046	0.0075
g_Roseburia	1	hsa-miR-103a-3p	0.784	-0.055	0.0072	0.8810
	2	hsa-miR-202-3p	0.768	-0.012	0.0094	0.9734
	3	hsa-miR-555	0.818	-0.297	0.0038	0.4047
	4	hsa-miR-627-3p	0.796	-0.492	0.0058	0.1482
	5	hsa-miR-766-5p	0.818	0.748	0.0038	0.0129

Table S6. A list of correlation coefficietns between six miRNAs and seven genera

		Correlation coefficient		p-value	
miRNA	Genus	MDD	HC	MDD	HC
hsa-miR-1246	*g_Anaerostipes*	-0.608	0.407	0.062	0.243
	g_Bacteroides	0.191	-0.188	0.597	0.602
	g_Bifidobacterium	-0.567	0.450	0.088	0.192
	g_Clostridium	0.533	0.547	0.113	0.102
	g_Collinsella	-0.294	0.535	0.410	0.111
	g_Dialister	0.356	-0.182	0.312	0.614
	g_Roseburia	0.137	0.024	0.707	0.947
hsa-miR-1276	*g_Anaerostipes*	0.019	0.558	0.959	0.093
	g_Bacteroides	0.598	0.178	0.068	0.623
	g_Bifidobacterium	-0.274	-0.276	0.443	0.440
	g_Clostridium	0.268	0.497	0.454	0.144
	g_Collinsella	-0.773	-0.276	0.009	0.440
	g_Dialister	0.075	-0.656	0.837	0.039
	g_Roseburia	-0.474	-0.043	0.167	0.906
hsa-miR-1976	*g_Anaerostipes*	0.442	0.188	0.200	0.603
	g_Bacteroides	0.115	-0.588	0.751	0.074
	g_Bifidobacterium	0.661	0.709	0.038	0.022
	g_Clostridium	-0.661	0.285	0.038	0.425
	g_Collinsella	0.273	0.721	0.446	0.019
	g_Dialister	0.030	0.261	0.934	0.467
	g_Roseburia	0.055	-0.212	0.881	0.556
hsa-miR-3144-3p	*g_Anaerostipes*	0.200	0.200	0.580	0.580
	g_Bacteroides	-0.115	-0.030	0.751	0.934
	g_Bifidobacterium	0.261	0.212	0.467	0.556
	g_Clostridium	-0.382	0.103	0.276	0.777
	g_Collinsella	0.055	-0.127	0.881	0.726
	g_Dialister	-0.717	-0.055	0.020	0.881
	g_Roseburia	-0.188	-0.673	0.603	0.033
hsa-miR-4488	*g_Anaerostipes*	-0.285	0.115	0.425	0.751
	g_Bacteroides	0.321	-0.285	0.365	0.425
	g_Bifidobacterium	-0.648	0.321	0.043	0.365
	g_Clostridium	0.042	0.103	0.907	0.777
	g_Collinsella	-0.176	0.515	0.627	0.128
	g_Dialister	0.036	-0.139	0.920	0.701
	g_Roseburia	-0.345	0.030	0.328	0.934
hsa-miR-579-3p	*g_Anaerostipes*	0.696	-0.015	0.025	0.967
	g_Bacteroides	-0.348	0.479	0.324	0.161
	g_Bifidobacterium	0.522	0.142	0.122	0.695
	g_Clostridium	0.087	-0.315	0.811	0.376
	g_Collinsella	0.087	0.225	0.811	0.532
	g_Dialister	-0.175	-0.689	0.629	0.027
	g_Roseburia	0.696	-0.255	0.025	0.478

Table S7. A list of negative correlation coefficients between miRNAs and genera in predicted miRNA functions

**1. Prion diseases (hsa05020)**	<1e-16	Genus	Correlation	p-value
hsa-miR-548h-5p|microT-CDS	2.71E-22	*Bacteroides*	-0.766	0.0098
hsa-miR-590-3p|microT-CDS	7.36E-14	*Bifidobacterium*	-0.770	0.0092
hsa-miR-30a-3p|microT-CDS	5.96E-40	*Collinsella*	-0.794	0.0061
**2. Transcriptional misregulation in cancer (hsa05202)**	<1e-16	Genus	Correlation	p-value
hsa-miR-338-5p|microT-CDS	4.44E-05	*Anaerostipes*	-0.766	0.0098
hsa-miR-548h-5p|microT-CDS	2.41E-06	*Bacteroides*	-0.766	0.0098
hsa-miR-1276|microT-CDS	8.43E-19	*Collinsella*	-0.773	0.0088
hsa-miR-30a-3p|microT-CDS	3.75E-05	*Collinsella*	-0.794	0.0061
hsa-miR-518d-3p|microT-CDS	5.04E-07	*Dialister*	-0.863	0.0013
hsa-miR-548l|microT-CDS	5.07E-05	*Roseburia*	-0.830	0.0029
**3. TGF-beta signaling pathway (hsa04350)**	9.97E-12	Genus	Correlation	p-value
hsa-miR-338-5p|microT-CDS	7.75E-05	*Anaerostipes*	-0.766	0.0098
hsa-miR-548h-5p|microT-CDS	4.44E-04	*Bacteroides*	-0.766	0.0098
hsa-miR-590-3p|microT-CDS	2.17E-08	*Bifidobacterium*	-0.770	0.0092
hsa-miR-374a-5p|microT-CDS	2.65E-12	*Collinsella*	-0.782	0.0075
hsa-miR-548l|microT-CDS	3.80E-06	*Roseburia*	-0.830	0.0029
**4. MAPK signaling pathway (hsa04010)**	4.90E-10	Genus	Correlation	p-value
hsa-miR-331-3p|microT-CDS	4.98E-10	*Anaerostipes*	-0.772	0.0089
hsa-miR-338-5p|microT-CDS	1.00E-06	*Anaerostipes*	-0.766	0.0098
hsa-miR-374a-5p|microT-CDS	1.29E-05	*Collinsella*	-0.782	0.0075
**5. Alcoholism (hsa05034)**	2.94E-09	Genus	Correlation	p-value
hsa-miR-1276|microT-CDS	1.78E-32	*Collinsella*	-0.773	0.0088
**6. Glycosaminoglycan biosynthesis - chondroitin sulfate (hsa00532)**	2.56E-08	Genus	Correlation	p-value
hsa-miR-338-5p|microT-CDS	8.01E-04	*Anaerostipes*	-0.766	0.0098
hsa-miR-548h-5p|microT-CDS	6.44E-08	*Bacteroides*	-0.766	0.0098
hsa-miR-4516|microT-CDS	2.27E-12	*Bifidobacterium*	-0.809	0.0046
hsa-miR-362-5p|microT-CDS	1.05E-12	*Collinsella*	-0.818	0.0038

**7. Ubiquitin mediated proteolysis (hsa04120)**	3.78E-08	Genus		Correlation	p-value
hsa-miR-548h-5p|microT-CDS	6.58E-11	*Bacteroides*		-0.766	0.0098
hsa-miR-590-3p|microT-CDS	9.64E-05	*Bifidobacterium*		-0.770	0.0092
hsa-miR-548l|microT-CDS	1.82E-04	*Roseburia*		-0.830	0.0029
**8. ECM-receptor interaction (hsa04512)**	1.54E-07	Genus		Correlation	p-value
hsa-miR-563|microT-CDS	3.63E-21	*Anaerostipes*		-0.794	0.0061
hsa-miR-196b-5p|microT-CDS	1.34E-04	*Bacteroides*		-0.766	0.0098
**9. Wnt signaling pathway (hsa04310)**	2.65E-07	Genus		Correlation	p-value
hsa-miR-548h-5p|microT-CDS	2.81E-05	*Bacteroides*		-0.766	0.0098
hsa-miR-891b|microT-CDS	7.48E-04	*Clostridium*		-0.782	0.0075
**10. Prostate cancer (hsa05215)**	7.66E-07	Genus		Correlation	p-value
hsa-miR-30a-3p|microT-CDS	3.75E-05	*Collinsella*		-0.794	0.0061
hsa-miR-182-5p|microT-CDS	9.48E-04	*Dialister*		-0.857	0.0015
**11. PI3K-Akt signaling pathway (hsa04151)**	1.27E-06	Genus		Correlation	p-value
hsa-miR-590-3p|microT-CDS	1.20E-06	*Bifidobacterium*		-0.770	0.0092
hsa-miR-182-5p|microT-CDS	2.34E-04	*Dialister*		-0.857	0.0015
**12. Focal adhesion (hsa04510)**	9.44E-06	Genus		Correlation	p-value
hsa-miR-590-3p|microT-CDS	9.52E-04	*Bifidobacterium*		-0.770	0.0092
hsa-miR-891b|microT-CDS	2.13E-04	*Clostridium*		-0.782	0.0075
hsa-miR-374a-5p|microT-CDS	9.84E-04	*Collinsella*		-0.782	0.0075
hsa-miR-92b-3p|microT-CDS	5.12E-05	*Roseburia*		-0.833	0.0029
**13. Adherens junction (hsa04520)**	2.57E-05	Genus		Correlation	p-value
hsa-miR-548h-5p|microT-CDS	6.73E-06	*Bacteroides*		-0.766	0.0098
hsa-miR-548l|microT-CDS	1.82E-04	*Roseburia*		-0.830	0.0029
**14. Systemic lupus erythematosus (hsa05322)**	6.57E-05	Genus		Correlation	p-value
hsa-miR-1276|microT-CDS	2.71E-27	*Collinsella*		-0.773	0.0088
**15. Biosynthesis of unsaturated fatty acids (hsa01040)**	9.26E-05	Genus		Correlation	p-value
hsa-miR-361-3p|microT-CDS	3.08E-13	*Anaerostipes*		-0.842	0.0022
hsa-miR-374a-5p|microT-CDS	4.86E-14	*Collinsella*		-0.782	0.0075

**16. Long-term potentiation (hsa04720)**	1.12E-04	Genus	Correlation	p-value
hsa-miR-338-5p|microT-CDS	4.78E-05	*Anaerostipes*	-0.766	0.0098
hsa-miR-590-3p|microT-CDS	3.94E-06	*Bifidobacterium*	-0.770	0.0092
hsa-miR-92b-3p|microT-CDS	3.76E-04	*Roseburia*	-0.833	0.0029
**17. Neurotrophin signaling pathway (hsa04722)**	2.26E-04	Genus	Correlation	p-value
hsa-miR-590-3p|microT-CDS	9.64E-05	*Bifidobacterium*	-0.770	0.0092
**18. Pathways in cancer (hsa05200)**	3.86E-04	Genus	Correlation	p-value
hsa-miR-374a-5p|microT-CDS	6.66E-08	*Collinsella*	-0.782	0.0075
**19. p53 signaling pathway (hsa04115)**	5.79E-04	Genus	Correlation	p-value
hsa-miR-590-3p|microT-CDS	4.84E-05	*Bifidobacterium*	-0.770	0.0092
hsa-miR-374a-5p|microT-CDS	2.10E-08	*Collinsella*	-0.782	0.0075
**20. Lysine degradation (hsa00310)**	6.97E-04	Genus	Correlation	p-value
hsa-miR-873-3p|microT-CDS	1.32E-07	*Clostridium*	-0.796	0.0058

Table S8. A list of positive correlation coefficients between miRNAs and genera in predicted miRNA functions

		Genus	Correlation	p-value
**1. Prion diseases (hsa05020)**	**<1e-16**	Genus	Correlation	p-value
hsa-miR-30e-3p|microT-CDS	6.60E-40	*Aanaerostipes*	0.881	0.0007
**2. Ubiquitin mediated proteolysis (hsa04120)**	**<1e-16**	Genus	Correlation	p-value
hsa-miR-3613-3p|microT-CDS	2.65E-34	*Bacteroides*	0.855	0.0016
hsa-miR-30b-5p|microT-CDS	4.76E-10	*Clostridium*	0.794	0.0061
**3. Prostate cancer (hsa05215)**	**<1e-16**	Genus	Correlation	p-value
hsa-miR-30e-3p|microT-CDS	3.90E-05	*Aanaerostipes*	0.881	0.0007
hsa-miR-320a|microT-CDS	1.11E-12	*Aanaerostipes*	0.806	0.0049
hsa-miR-3613-3p|microT-CDS	9.14E-09	*Bacteroides*	0.855	0.0016
hsa-miR-1185-1-3p|microT-CDS	9.54E-05	*Bifidobacterium*	0.794	0.0061
hsa-miR-212-3p|microT-CDS	4.20E-04	*Bifidobacterium*	0.891	0.0005
**4. PI3K-Akt signaling pathway (hsa04151)**	**5.65E-14**	Genus	Correlation	p-value
hsa-miR-320a|microT-CDS	3.91E-07	*Aanaerostipes*	0.806	0.0049
hsa-miR-3613-3p|microT-CDS	1.37E-07	*Bacteroides*	0.855	0.0016
hsa-miR-1185-1-3p|microT-CDS	5.89E-08	*Bifidobacterium*	0.794	0.0061
**5. Wnt signaling pathway (hsa04310)**	**6.75E-14**	Genus	Correlation	p-value
hsa-miR-3613-3p|microT-CDS	7.75E-19	*Bacteroides*	0.855	0.0016
**6. Transcriptional misregulation in cancer (hsa05202)**	**1.41E-12**	Genus	Correlation	p-value
hsa-miR-30e-3p|microT-CDS	3.90E-05	*Aanaerostipes*	0.881	0.0007
hsa-miR-3613-3p|microT-CDS	3.90E-15	*Bacteroides*	0.855	0.0016
hsa-miR-1185-1-3p|microT-CDS	1.56E-04	*Bifidobacterium*	0.794	0.0061
**7. Pathways in cancer (hsa05200)**	**3.15E-12**	Genus	Correlation	p-value
hsa-miR-320a|microT-CDS	1.50E-05	*Aanaerostipes*	0.806	0.0049
hsa-miR-3613-3p|microT-CDS	3.63E-14	*Bacteroides*	0.855	0.0016
hsa-miR-103a-3p|microT-CDS	7.69E-05	*Roseburia*	0.784	0.0072
**8. TGF-beta signaling pathway (hsa04350)**	**9.85E-12**	Genus	Correlation	p-value
hsa-miR-3613-3p|microT-CDS	3.04E-09	*Bacteroides*	0.855	0.0016
hsa-miR-1185-1-3p|microT-CDS	5.16E-08	*Bifidobacterium*	0.794	0.0061
hsa-miR-212-3p|microT-CDS	8.65E-07	*Bifidobacterium*	0.891	0.0005
**9. Focal adhesion (hsa04510)**	**1.24E-09**	Genus	Correlation	p-value
hsa-miR-320a|microT-CDS	6.88E-06	*Aanaerostipes*	0.806	0.0049
hsa-miR-3613-3p|microT-CDS	5.46E-06	*Bacteroides*	0.855	0.0016
hsa-miR-1185-1-3p|microT-CDS	1.75E-04	*Bifidobacterium*	0.794	0.0061
**10. Neurotrophin signaling pathway (hsa04722)**	**1.95E-09**	Genus	Correlation	p-value
hsa-miR-3613-3p|microT-CDS	1.18E-05	*Bacteroides*	0.855	0.0016
hsa-miR-1185-1-3p|microT-CDS	3.61E-04	*Bifidobacterium*	0.794	0.0061
hsa-miR-212-3p|microT-CDS	4.20E-04	*Bifidobacterium*	0.891	0.0005
hsa-miR-103a-3p|microT-CDS	8.29E-04	*Roseburia*	0.784	0.0072
**11. Adherens junction (hsa04520)**	**1.99E-09**	Genus	Correlation	p-value
hsa-miR-3613-3p|microT-CDS	3.27E-08	*Bacteroides*	0.855	0.0016
hsa-miR-544a|microT-CDS	1.12E-09	*Collinsella*	0.766	0.0098
**12. Axon guidance (hsa04360)**	**2.74E-09**	Genus	Correlation	p-value
hsa-miR-3613-3p|microT-CDS	3.04E-09	*Bacteroides*	0.855	0.0016
hsa-miR-212-3p|microT-CDS	9.64E-04	*Bifidobacterium*	0.891	0.0005
hsa-miR-30b-5p|microT-CDS	4.09E-08	*Clostridium*	0.794	0.0061
**13. Circadian rhythm (hsa04710)**	**3.38E-09**	Genus	Correlation	p-value
hsa-miR-320a|microT-CDS	6.84E-06	*Aanaerostipes*	0.806	0.0049
hsa-miR-3613-3p|microT-CDS	1.01E-10	*Bacteroides*	0.855	0.0016
hsa-miR-346|microT-CDS	2.01E-06	*Dialister*	0.790	0.0065
**14. Regulation of actin cytoskeleton (hsa04810)**	**1.03E-08**	Genus	Correlation	p-value
hsa-miR-3613-3p|microT-CDS	8.36E-07	*Bacteroides*	0.855	0.0016
hsa-miR-1185-1-3p|microT-CDS	2.56E-05	*Bifidobacterium*	0.794	0.0061
hsa-miR-4425|microT-CDS	1.19E-06	*Bifidobacterium*	0.855	0.0016

**15. Colorectal cancer (hsa05210)**	**1.57E-08**	Genus	Correlation	p-value
hsa-miR-320a|microT-CDS	6.48E-05	*Aanaerostipes*	0.806	0.0049
hsa-miR-3613-3p|microT-CDS	9.36E-10	*Bacteroides*	0.855	0.0016
hsa-miR-1185-1-3p|microT-CDS	7.77E-05	*Bifidobacterium*	0.794	0.0061
**16. MAPK signaling pathway (hsa04010)**	**2.40E-08**	Genus	Correlation	p-value
hsa-miR-3613-3p|microT-CDS	2.86E-07	*Bacteroides*	0.855	0.0016
hsa-miR-1185-1-3p|microT-CDS	7.03E-05	*Bifidobacterium*	0.794	0.0061
**17. Renal cell carcinoma (hsa05211)**	**3.47E-08**	Genus	Correlation	p-value
hsa-miR-30e-3p|microT-CDS	3.91E-05	*Aanaerostipes*	0.881	0.0007
hsa-miR-320a|microT-CDS	1.28E-05	*Aanaerostipes*	0.806	0.0049
hsa-miR-3613-3p|microT-CDS	1.24E-08	*Bacteroides*	0.855	0.0016
hsa-miR-212-3p|microT-CDS	1.96E-05	*Bifidobacterium*	0.891	0.0005
**18. Shigellosis (hsa05131)**	**5.39E-08**	Genus	Correlation	p-value
hsa-miR-3613-3p|microT-CDS	2.70E-05	*Bacteroides*	0.855	0.0016
hsa-miR-1185-1-3p|microT-CDS	8.79E-04	*Bifidobacterium*	0.794	0.0061
hsa-miR-212-3p|microT-CDS	1.89E-04	*Bifidobacterium*	0.891	0.0005
hsa-miR-4425|microT-CDS	4.91E-07	*Bifidobacterium*	0.855	0.0016
**19. Endometrial cancer (hsa05213)**	**6.27E-08**	Genus	Correlation	p-value
hsa-miR-320a|microT-CDS	2.26E-10	*Aanaerostipes*	0.806	0.0049
hsa-miR-212-3p|microT-CDS	5.23E-05	*Bifidobacterium*	0.891	0.0005
**20. Fatty acid biosynthesis (hsa00061)**	**2.56E-07**	Genus	Correlation	p-value
hsa-miR-103a-3p|microT-CDS	4.76E-25	*Roseburia*	0.784	0.0072
**21. Lysine degradation (hsa00310)**	**7.94E-07**	Genus	Correlation	p-value
hsa-miR-654-3p|microT-CDS	4.08E-08	*Clostridium*	0.782	0.0075
**22. ErbB signaling pathway (hsa04012)**	**1.33E-06**	Genus	Correlation	p-value
hsa-miR-1185-1-3p|microT-CDS	3.04E-06	*Bifidobacterium*	0.794	0.0061
hsa-miR-671-3p|microT-CDS	1.42E-05	*Dialister*	0.790	0.0065
**23. Insulin signaling pathway (hsa04910)**	**2.37E-06**	Genus	Correlation	p-value
hsa-miR-320a|microT-CDS	3.95E-04	*Aanaerostipes*	0.806	0.0049
hsa-miR-3613-3p|microT-CDS	3.71E-04	*Bacteroides*	0.855	0.0016
**24. mTOR signaling pathway (hsa04150)**	**4.43E-06**	Genus	Correlation	p-value
hsa-miR-320a|microT-CDS	7.13E-08	*Aanaerostipes*	0.806	0.0049
hsa-miR-103a-3p|microT-CDS	1.69E-04	*Roseburia*	0.784	0.0072
**25. Chronic myeloid leukemia (hsa05220)**	**8.10E-06**	Genus	Correlation	p-value
hsa-miR-320a|microT-CDS	3.28E-04	*Aanaerostipes*	0.806	0.0049
hsa-miR-3613-3p|microT-CDS	9.23E-08	*Bacteroides*	0.855	0.0016
hsa-miR-212-3p|microT-CDS	9.69E-06	*Bifidobacterium*	0.891	0.0005
**26. Pancreatic cancer (hsa05212)**	**9.16E-06**	Genus	Correlation	p-value
hsa-miR-3613-3p|microT-CDS	3.13E-05	*Bacteroides*	0.855	0.0016
**27. Non-small cell lung cancer (hsa05223)**	**9.35E-06**	Genus	Correlation	p-value
hsa-miR-320a|microT-CDS	2.04E-06	*Aanaerostipes*	0.806	0.0049
hsa-miR-212-3p|microT-CDS	9.69E-06	*Bifidobacterium*	0.891	0.0005
**28. Hepatitis B (hsa05161)**	**3.88E-05**	Genus	Correlation	p-value
hsa-miR-3613-3p|microT-CDS	2.43E-05	*Bacteroides*	0.855	0.0016
hsa-miR-1185-1-3p|microT-CDS	6.36E-05	*Bifidobacterium*	0.794	0.0061
**29. Long-term potentiation (hsa04720)**	**5.53E-05**	Genus	Correlation	p-value
hsa-miR-363-3p|microT-CDS	4.23E-05	*Bifidobacterium*	0.848	0.002
hsa-miR-30b-5p|microT-CDS	2.02E-04	*Clostridium*	0.794	0.0061
**30. Protein processing in endoplasmic reticulum (hsa04141)**	**6.39E-05**	Genus	Correlation	p-value
hsa-miR-3613-3p|microT-CDS	3.04E-09	*Bacteroides*	0.855	0.0016
hsa-miR-30b-5p|microT-CDS	3.26E-04	*Clostridium*	0.794	0.0061
**31. B cell receptor signaling pathway (hsa04662)**	**7.05E-05**	Genus	Correlation	p-value
hsa-miR-212-3p|microT-CDS	6.37E-04	*Bifidobacterium*	0.891	0.0005
hsa-miR-30b-5p|microT-CDS	1.42E-07	*Clostridium*	0.794	0.0061

**32. Glioma (hsa05214)**	**7.15E-05**	Genus	Correlation	p-value
hsa-miR-320a|microT-CDS	2.21E-06	*Aanaerostipes*	0.806	0.0049
hsa-miR-1185-1-3p|microT-CDS	8.54E-04	*Bifidobacterium*	0.794	0.0061
hsa-miR-212-3p|microT-CDS	9.64E-04	*Bifidobacterium*	0.891	0.0005
**33. mRNA surveillance pathway (hsa03015)**	**9.79E-05**	Genus	Correlation	p-value
hsa-miR-3613-3p|microT-CDS	6.26E-09	*Bacteroides*	0.855	0.0016
**34. Small cell lung cancer (hsa05222)**	**9.88E-05**	Genus	Correlation	p-value
hsa-miR-3613-3p|microT-CDS	1.11E-06	*Bacteroides*	0.855	0.0016
**35. HTLV-I infection (hsa05166)**	**1.53E-04**	Genus	Correlation	p-value
hsa-miR-3613-3p|microT-CDS	7.62E-05	*Bacteroides*	0.855	0.0016
**36. Hypertrophic cardiomyopathy (HCM) (hsa05410)**	**1.94E-04**	Genus	Correlation	p-value
hsa-miR-654-3p|microT-CDS	1.11E-04	*Clostridium*	0.782	0.0075
**37. Dopaminergic synapse (hsa04728)**	**5.54E-04**	Genus	Correlation	p-value
hsa-miR-3613-3p|microT-CDS	3.90E-04	*Bacteroides*	0.855	0.0016
hsa-miR-1185-1-3p|microT-CDS	3.60E-04	*Bifidobacterium*	0.794	0.0061
